# Loss-of-Function Screen Reveals Novel Regulators Required for *Drosophila* Germline Stem Cell Self-Renewal

**DOI:** 10.1534/g3.111.001651

**Published:** 2012-03-01

**Authors:** Yalan Xing, Irina Kurtz, Manisha Thuparani, Jillian Legard, Hannele Ruohola-Baker

**Affiliations:** Department of Biochemistry, Institute for Stem Cell & Regenerative Medicine, University of Washington, Seattle, Washington 98109

**Keywords:** loss-of-function screen, *Drosophila* germline stem cell (GSC), ubiquitin-conjugating enzyme, *pineapple eye* (*pie*)

## Abstract

The germline stem cells (GSCs) of *Drosophila melanogaster* ovary provide an excellent model system to study the molecular mechanisms of stem cell self-renewal. To reveal novel factors required for *Drosophila* female GSC maintenance and/or division, we performed a loss-of-function screen in GSCs by using a collection of *P*-element–induced alleles of essential genes. Mutations in genes of various functional groups were identified to cause defects in GSC self-renewal. Here we report that a group of mutations affecting various ubiquitin-conjugating enzymes cause significant GSCs loss, including *Plenty of SH3s* (*POSH*), *Ubiquitin-conjugating enzyme 10* (*UbcD10*), and *pineapple eye* (*pie*). Ubiquitin-mediated protein degradation plays a variety of roles in the regulation of many developmental processes, including mediating stem cell division through degradation of cell cycle regulators. We demonstrated that *pie*, sharing highly conserved RING domains with human E3 ubiquitin ligase G2E3 that are critical for early embryonic development, is specifically required for GSC maintenance, possibly through regulation of bone morphogenetic protein signaling pathway. Despite the previously reported role in imaginal disc cell survival, *pie* loss-of-function induced GSC loss is not to the result of caspase-involved cell death. Further efforts are needed to elucidate the functions of ubiquitin ligases in GSC maintenance, which will ultimately contribute to a better understanding of how the ubiquitin-conjugating enzymes regulate stem cell biology in mammalian systems.

The germline stem cells (GSCs) of the *Drosophila* ovary provide an excellent model system for studying the mechanisms of adult stem cell self-renewal (Lin 1997; Yamashita *et al.* 2005; Fuller and Spradling 2007). Two to three GSCs, marked by spherical spectrosomes, locate at the apical tip of a *Drosophila* ovary germarium, in direct contact with the somatic niche composed of terminal filaments and cap cells (Figure 1A). In an asymmetrical division, the GSC divides along the anteroposterior axis to the niche, producing a GSC and a daughter cystblast to the posterior direction. After four incomplete divisions, the cystoblast becomes a cyst with 16 interconnected cells (Lin 2002).

**Figure 1:**
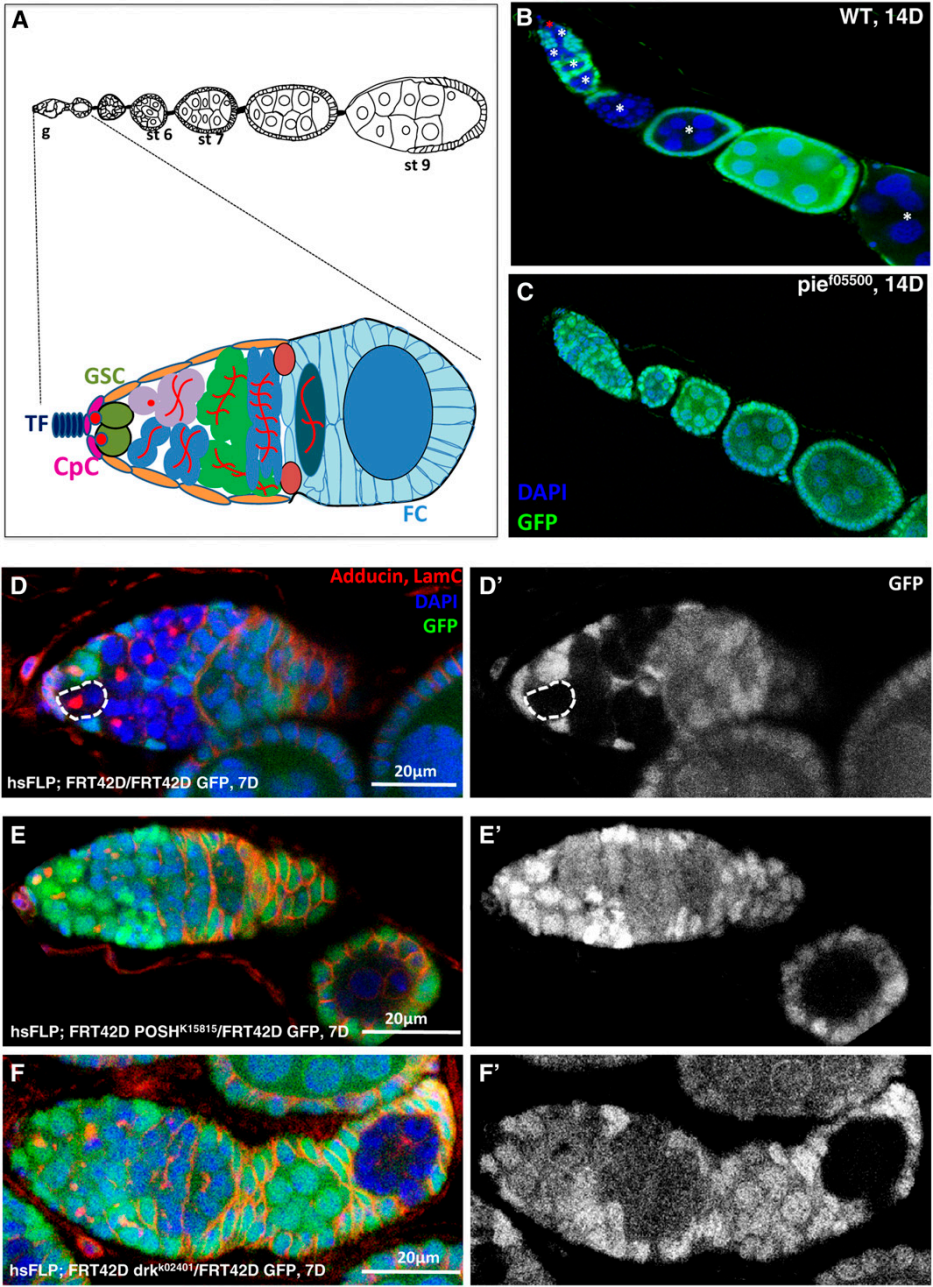
Loss-of-function screen reveals key factors required for GSC maintenance. (A) Schematic diagrams of the *Drosophila* ovariole and the germarium. Different cell types are indicated as follows: terminal filament (TF), cap cells (CpC), germline stem cells (GSC), and follicle cells (FC). Red dots and branched structures represent spectrosomes. (B) Wild-type clonal GSCs continuously make progenies 14 days after heat shock induction (GFP−, asterisks). (C) None of *pie*−/− clonal GSCs or progenies are observed in an ovariole 14 days after heat shock induction (lack of GFP− cysts). (D, D´) Wild-type clonal GSC (GFP−, dashed circle) is still maintained in the niche 7 days after induction. (E, E´, F, F´) *POSH−/−* and *drk−/−* clonal GSCs are lost within 7 days, and the latest progeny developed into a stage5 egg chamber (GFP−).

Self-renewal of female GSCs is primarily regulated by bone morphogenetic protein (BMP) signaling from the niche, mediated by the ligands decapentaplegic (*dpp*) and glassbottomed boat (*gbb*) (Xie and Spradling 1998; Song *et al.* 2004). Notch activity in the niche cells is shown to regulate this process (Ward *et al.* 2006). The BMP signaling activates cytoplasmic Mad and Medea, the *Drosophila* Smads, in GSCs, which form complex and silence the transcription of *bag-of-marble (bam)* gene, the key differentiation factor that is normally turned off in GSCs (Chen and McKearin 2003a; Song *et al.* 2004). In addition to the extrinsic regulatory signaling from the niche, GSC self-renewal is also mediated by intrinsic factors, which repress GSC differentiation. Pumilio (Pum)/Nanos (Nos) complex-mediated or microRNA-mediated translational repression is shown to be essential for GSC maintenance and division (Gilboa and Lehmann 2004; Hatfield *et al.* 2005; Szakmary *et al.* 2005; Shcherbata *et al.* 2007).

To reveal novel factors required for GSC self-renewal, we performed a loss-of-function screen in GSCs, which yielded a group of mutations affecting various ubiquitin-conjugating enzymes, including *plenty of SH3s* (*POSH*), *ubiquitin-conjugating enzyme 10* (*UbcD10*), and *pineapple eye* (*pie*). Ubiquitin-mediated posttranslational regulation plays a variety of roles in many developmental processes, including the maintenance of quiescence in stem cells (Onoyama and Nakayama 2008; Clague and Urbe 2010). In the following studies, we will demonstrate a nonapoptotic role of *pie*, which was previously reported as a survival factor (Shi *et al.* 2003), in GSC maintenance.

## Materials and Methods

### Fly stocks and culture conditions

The following stocks and other deficiency alleles were obtained from *Drosophila* Genetic Resource Center, Kyoto Institute of Technology, Japan: *y^d2^w^1118^P{ey-FLP.N}2*; *PBac{WH} pie^f05500^ P{neoFRT}40A/CyO*, *y w1118 P{ey-FLP.N}2 P{GMR-lacZ.C(38.1)}TPN1*; *P{neoFRT}42D P{lacW}POSH^k15815^ /CyO y+*, *yd2 w1118 P{ey-FLP.N}2 P{GMR-lacZ.C(38.1)}TPN1*; *P{neoFRT}42D P{GT1}UbcD10^BG00902^ /CyO y+*, *yd2 w1118 P{ey-FLP.N}2 P{GMR-lacZ.C(38.1)}TPN1*; *P{lacW}kek1^k07332^ P{neoFRT}40A/CyO y+*, *y[d2] w[1118] P{ry[+t7.2]=ey-FLP.N}2 P{GMR-lacZ.C(38.1)}TPN1*; *P{w[+mC]=lacW}ebi^[k16213]^ P{ry[+t7.2]=neoFRT}40A/CyO y[+],w[1118]*; *P{ry[+t7.2]=neoFRT}42D P{w[+mC]=lacW}drk^[k02401]^ /CyO y[+]*, *FRT42BdrkΔP24/CyO*, *yd2 w1118 P{ey-FLP.N}2 P{GMR-lacZ.C(38.1)}TPN1*; *P{lacW}ab^k02807^ P{neoFRT}40A/CyO y+*, *yd2 w1118 P{ey-FLP.N}2 P{GMR-lacZ.C(38.1)}TPN1*; *P{SUPor-P}crp^KG08234^ P{neoFRT}40A/CyO y+*, *yd2 w1118 P{ey-FLP.N}2 P{GMR-lacZ.C(38.1)}TPN1*; *P{lacW}l(2)k14505^k14505^ P{neoFRT}40A/CyO y+*, *yd2 w1118 P{ey-FLP.N}2 P{GMR-lacZ.C(38.1)}TPN1*; *P{lacW}Df31^k05815^ P{neoFRT}40A/CyO y+*, *yd2 w1118 P{ey-FLP.N}2 P{GMR-lacZ.C(38.1)}TPN1*; *P{lacW}snRNA:U6atac:29B^k01105^ P{neoFRT}40A/CyO y+*.

The following stocks are obtained from Bloomington Drosophila Stock Center at Indiana University: *w*; *FRT40ApieE1-16/SM5-TM6*, *y+*, *hsFLP;Ubi-GFP FRT40A/CyO*, *hsFLP;arm-LacZ FRT40A/CyO w;FRT40A/CyO*, *hsFLP;FRT42BUbi-GFP/CyO*, *hsFlp;FRT42DUbi-GFP/CyO*, *w*; *pin/cyo;Ly/TM6B*. *w;;bam-GFP* was a generous gift from the McKearin lab. Flies were cultured at 25° on standard cornmeal−yeast−agar medium (protein-rich diet). For starvation assay, females of desired genotypes were fed with a protein-rich diet or in an empty vial containing a Kimwipe soaked in 10% light corn syrup (protein-poor diet) for 5 days at 25° before analysis.

### Immunofluorescence and microscopy

Ovaries of desired genotypes were dissected in phosphate-buffered saline (PBS) and immediately fixed in PBS containing 4% paraformaldehyde and then stained as described (Shcherbata *et al.* 2004). The following primary antibodies were used: mouse anti-adducin, mouse anti-Lamin C (Developmental Studies Hybridoma Bank, 1:20); rabbit anticleaved caspase 3 and rabbit anti-pMad (Cell Signaling Technology, 1:250 and 1:50); and rabbit anti-βgal 1:5000. Secondary fluorescence antibodies used include Alexa 488, 568, and 633 antimouse or antirabbit (1:250). Samples were mounted and analyzed on Leica SPE5 and Nikon N1 confocal laser-scanning microscopes.

### Terminal deoxynucleotidyl transferase dUTP nick end labeling (TUNEL) assay

The *In Situ* Cell Death Detection Kit (Roche Applied Science) was used as an independent means of detecting apoptotic cells (Sgonc *et al.* 1994). Ovaries were dissected in cold PBS, fixed in 4% paraformaldehyde, and processed according to manufacturer’s protocol. Labeled ovaries were counterstained with DAPI, mounted, and analyzed with fluorescent microscope.

### Generation of clones by FLP-mediated mitotic recombination

Two- to four-day-old females or males of the following genotypes *hsFLP;Ubi-GFP FRT40A/GFP FRT40A*, *hsFLP*; *Ubi-GFP FRT40A/ pie^E1-16^ FRT40A*, *hsFLP;Ubi-GFP FRT40A/PBac{WH}pie^f05500^ P{neoFRT}40A*, *hsFLP;Ubi-GFP FRT40A/GFP FRT40A;UASp-p35/nosGAL4*, *hsFLP;Ubi-GFP FRT40A/PBac{WH}pie^f05500^ P{neoFRT}40A;UASp-p35/nosGAL4*, *hsFLP*; *arm-LacZ FRT40A/ pie^E1-16^ FRT40A*; *bam-GFP/+* were heat shocked for 45 min in a 37° water bath for 2 consecutive days to induce mitotic recombination. Heat shocked flies were kept at 25° and transferred to fresh food with wet yeast paste every other day before dissection.

### Maintenance analysis

The GSC loss per day was calculated by comparing % of germaria with clonal GSCs at two time points after the heat-shock induction: GSC loss per day = (% of germaria with clonal GSCs at day 5 − % of clonal germaria at day 11)/% of clonal germaria at day 5/6 days (Shcherbata *et al.* 2007). All results were subjected to Student’s *t*-test.

### Loss of function screen for genes that affect *Drosophila* germline GSC maintenance and division

For primary screen, third instar larvae were heat shocked for 60 min in a 37° water bath for 2 consecutive days and dissected 14 days after the last heat shock. Ovaries were analyzed for the presence of clonal (GFP-negative) egg chambers. For secondary screen, 2-4 day old female adults were heat shocked for 45 min in 37° water bath for 2 consecutive days and dissected at 7 and 14 days after induction.

## Results

### Genetic screen for key regulators in *Drosophila* female GSC maintenance

To reveal novel factors required for *Drosophila* female GSC maintenance and division, we performed a loss-of-function screen by using 820 *P*-element induced mutant lines of essential genes (*Drosophila* Genetic Resource Center DGRC, Kyoto). Each *P*-element line recombined with FRT foci was crossed to corresponding FRT, Ubi-GFP line; then, homozygous clones were generated through heat shock flippase mediated mitotic recombination in young adult females (*Materials and Methods*, supporting information, Table S1). The primary screen yielded 43 positive lines on the basis of the phenotype that no or very few homozygous clones (GFP−) were recovered in the ovarioles 14 days after heat shock induction (Figure 1C), indicating a defect in GSC maintenance or division. In contrast, the negative control lines displayed homozygous clones all along the ovarioles, suggesting that GSCs are still properly producing progenies (Figure 1B).

**Figure 2:**
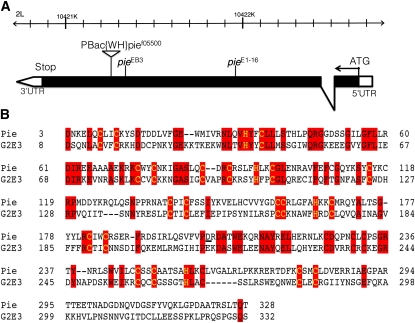
*pie* shares high similarity with human G2E3 RING domains. (A) Genomic map of *pie* shows locations of various mutant lines used. *pie^E1-16^* and *pie^EB3^* are ethyl-methanesulfonate-induced loss-of-function mutants; *pie^f05500^* is a P-element insertion-caused loss-of-function line. (B) Amino acid sequence alignment of *pie* protein N-terminal and human G2E3 RING domains. Red blocks indicate identical amino acids. The null allele *pie^E1-16^* is caused by frame shift at Asp203 (underlined).

In the secondary screen, each candidate line was further examined by assessing the rate of mutant GSC clone loss between 7 and 14 days after clonal induction (see *Materials and Methods*). Ten lines of 43 candidate mutant lines from the primary screen were confirmed to result in rapid GSC loss after clone induction in the secondary screen (Figure 1, D−F; Table 1). Functions of genes affected by these 10 lines fall into various categories spanning from posttranslational modification, epidermal growth factor receptor signaling pathway, to transcription factors, chromatin regulators, and mitochondria activity. A group of genes, including *POSH*, *UbcD10*, and *pie*, came to our interest. *POSH* and *UbcD10*, respectively, encode for *Drosophila* E3 and E2 ubiquitin ligase (Figure 2B) (Tsuda *et al.* 2005; Yatsenko *et al.* 2007); whereas *pie*, with predicted RING/PHD domains, shares highly conserved sequence with a human E3 ubiquitin ligase G2E3 (Shi *et al.* 2003; Brooks *et al.* 2008) The authors of a recent study have demonstrated that another ubiquitin-conjugating enzyme, Effete, maintains *Drosophila* GSCs through regulating cyclin A degradation (Chen *et al.* 2009). The finding of this group of ubiquitin ligase genes to be essential for GSC maintenance suggests a potentially conserved role of ubiquitin-mediated proteolysis in stem cell self-renewal and tissue homeostasis.

**Table 1:** Deficiency screen for novel regulators in GSC self-renewal

Function Groups	Gene Name	Allele(s)	Function	% Clonal Day14*^a^*	GSC Loss/Day*^b^*	Passed 2′ Screen
I. Ubiquitin ligase	*POSH*	hsFLP; FRT42DPOSH^k15815^/FRT42DGFP	E3 ubiquitin ligase	0%	30%	Yes
	*Pie*	hsFLP; pie^E1-16^FRT40A/FRT40AGFP	E3 ubiquitin ligase (Predicted)	0%	28.76%	Yes
		hsFLP; pie^f05500^ FRT40A/FRT40AGFP		0%	25%	
	*UbcD10*	hsFLP; FRT42DUbcD10^BG00902^/FRT42DGFP	E2 ubiquitin ligase	3.40%	/	Yes
II. EGFR signaling	*Kekkon*-*1*	hsFLP; kek1^k07332^ FRT40A/FRT40AGFP	Repressor of EGFR signaling	11%	9%	Yes
	*drk*	hsFLP; FRT42Ddrk^k02401^/FRT42DGFP	RTK adaptor protein	4.80%	7.59%	Yes
		hsFLP; FRT42Bdrk^ΔP24^/FRT42BGFP		25.00%	4.08%	
III. Transcription factors	*Abrupt*	hsFLP; ab^k02807^ FRT40A/ FRT40AGFP	Transcription factor activity Specific RNA polymerase II	6.9%	10.5%	Yes
	*Cropped*	hsFLP; crp^KG08234^FRT40A/FRT40AGFP	Transcription factor	0%	28.7%	Yes
IV. Housekeeping genes	*CG8674*	hsFLP; k14505^k14505^FRT40A/FRT40AGFP	Proton-transporting ATP synthase complex assembly	0%	/	Yes
	*Df31*	hsFLP; Df31^k05815^FRT40A/FRT40AGFP	chromatin organization	0%	/	Yes
	*snRNA:U6atac:29B*	hsFLP;snRNA:U6atac:29B^k01105^FRT40A/ FRT40AGFP	nuclear mRNA splicing	6%	/	Yes

EGFR, epidermal growth factor receptor; GSC, germline stem cell; RTK, receptor tyrosine kinase.

a &.

b n > 80.

### *Pie* is cell-autonomously required for *Drosophila* female GSC maintenance

**Figure 3:**
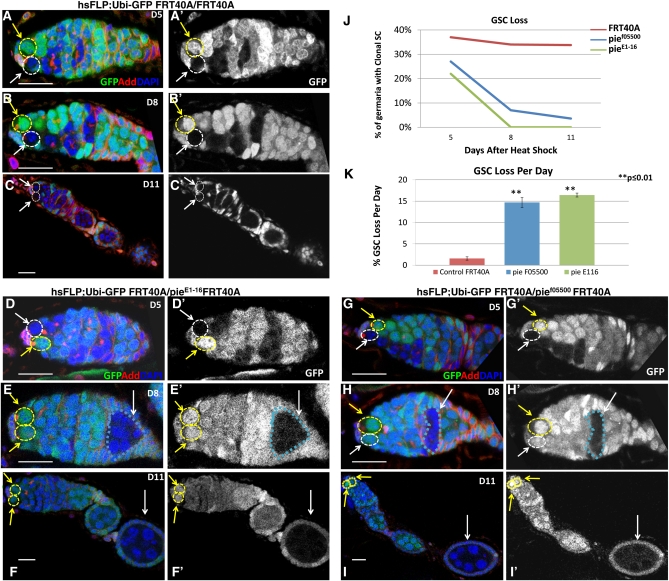
*pie* is cell-autonomously required for GSC maintenance. (A–I) Ovaries from *hsFlp*; *Ubi-GFP FRT40A/FRT40A* control (A−C), *hsFlp*; *Ubi-GFP FRT40A/pie^E1-16^ FRT40A* (D−F), and *hsFlp*; *Ubi-GFP FRT40A/pie^f05500^ FRT40A* (G−I) flies were collected at indicated days after the heat shock induction and labeled with Adducin (red, spectrosomes), Lamin C (red, cap cell nuclear envelope), and DAPI. Scale bar = 20 μm. (A−C) Clonal GSCs, marked by the absence of GFP (white arrows), and nonclonal GSCs (yellow arrows) are present in control germaria at 5, 8, and 11 days after clonal induction. (D−I) *Pie* clonal homozygous mutant GSCs are present in anterior niche at 5 days after heat-shock (white dashed circle) but are missing at 8 and 11 days after heat shock treatment. *pie* mutant GSCs are capable of developing into cysts and egg chambers and this can be observed 8 and 11 days after heat shock (white arrows and blue circles). Yellow arrows indicate nonclonal GSCs. (J) This graph summarizes changes in % of clonal germaria over time in control, *pie^f05500^*, and *pie^E1-16^* flies after one experiment. (K) The percentages of mutant GSC loss per day (see *Materials and Methods*) are significantly greater for both *pie^f05500^* and *pie^E1-16^* alleles, compared to FRT40A control. ^**^P < 0.01, Student’s *t*-test.

The *Drosophila pineapple eye (pie)* gene, located on the left arm of the 2nd chromosome, was previously identified to encode a novel 582-amino acid protein essential for imaginal disc cell survival (Figure 2A) (Shi *et al.* 2003). To further explore whether *pie* is cell-autonomously required for female GSC maintenance, we generated homozygous loss-of-function clones in young females by using two different alleles, a hypomorphic mutant *pie^E1-16^* and a piggyBac transposase-induced mutant *pie^f05500^*, and examined germaria at serial time points (5, 8, and 11 days) after heat shock induction (*Materials and Methods*). Compared with wild-type GSC clones, GSC clones mutant for either allele of *pie* were frequently recovered at day 5, but rapidly lost with time (Figure 3; Table 2). Notably, although clonal GSCs are rarely seen after Day 8, we were still able to recover clonal differentiated cysts frequently (Figure 3, E and H, blue dashed circles and white arrows). This evidence indicates that *pie*-mutant GSCs fail to self-renew, leave the niche, and enter differentiation.

**Table 2:** Pie is required for GSC maintenance

Genotype	% of Germaria With Clonal GSC	% of Germaria With Clonal GSC			Average Loss per day± SD
		5 Day	8 Day	11 Day	
*Control hsFLP*; *Ubi-GFP FRT40A/FRT 40A*	Exp. I	37% (n = 112)	34% (n = 44)	33.8% (n = 62)	1.57 ± 0.39
	Exp. II	38% (n = 100)	34% (n = 74)	35% (n = 100)	
	Exp. III	33% (n = 21)	31% (n = 38)	29% (n = 32)	
*pie^E1-16^ hsFLP*; *Ubi-GFP FRT40A/pie^E1-16^ 40A*	Exp. I	22% (n = 90)	0% (n = 62)	0% (n = 74)	16.43 ± 0.46
	Exp. II	23% (n = 100)	9% (n = 115)	1% (n = 87)	
	Exp. III	11% (n = 84)	0% (n = 80)	0% (n = 73)	
*pie^F05500^ hsFLP*; *Ubi-GFP FRT40A/pie^F05500^ 40A*	Exp. I	27% (n = 85)	7% (n = 90)	3.6% (n = 55)	14.7 ± 1.18
	Exp. II	34% (n = 100)	8% (n = 91)	6% (n = 47)	
	Exp. III	27% (n = 62)	5% (n = 80)	1% (n = 100)	

### Cell death is not responsible for *pie* mutant GSC maintenance defects

**Figure 4:**
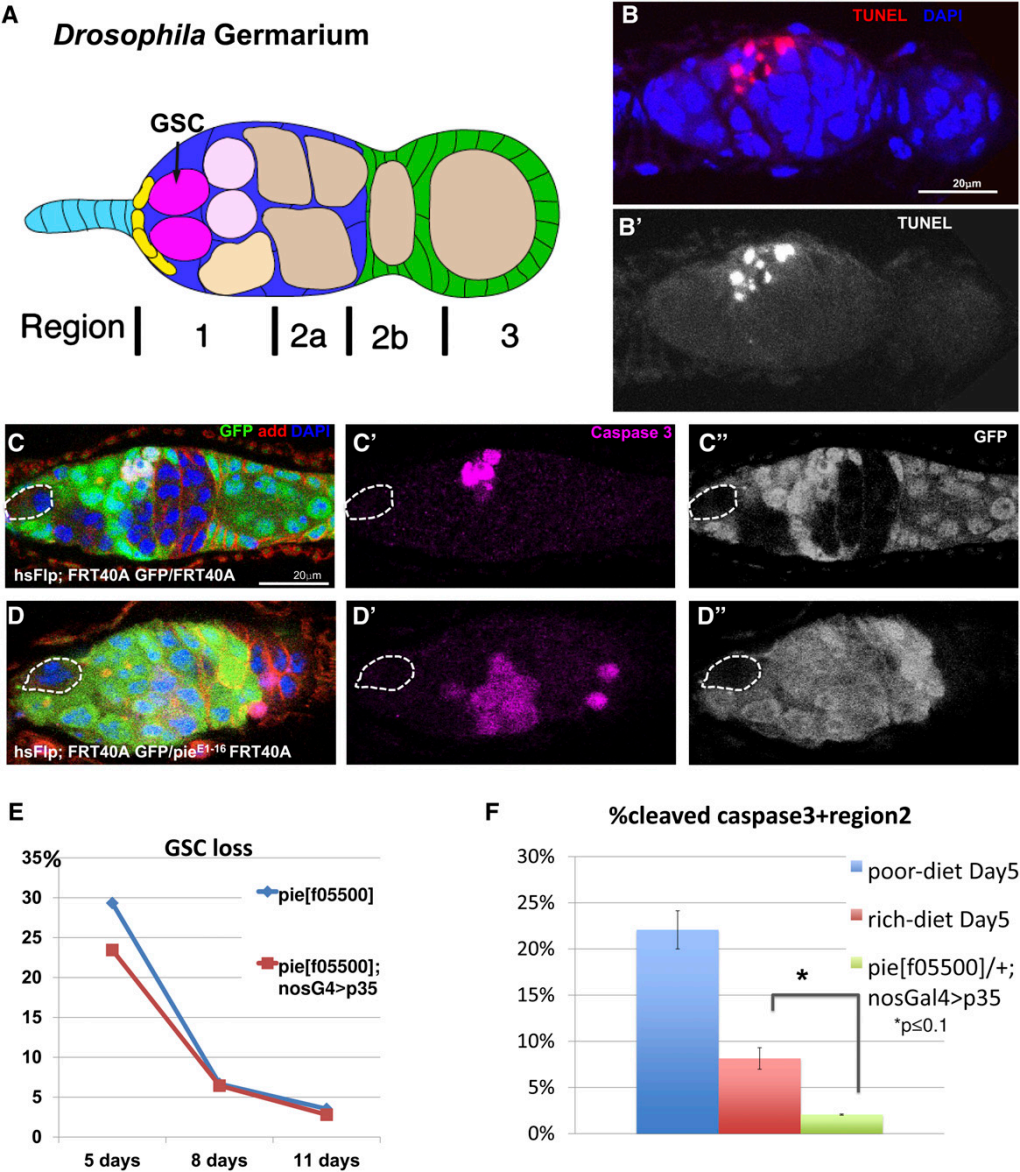
*pie* is not required for GSC survival. (A) *Drosophila* germarium cysts categorized by region 1−3. (B and B′). TUNEL assay recognizes cell death in region 2 cysts upon 5 days of protein-depleted diet (method). (C and D) At 8 days after heat shock induction, neither wild-type GSCs (C, dashed circle) nor *pie^E1-16^* GSCs (D, dashed circle) are positive for cleaved caspase 3 (magenta, cell death marker), whereas in both cases, there is a basal frequency of cell death in region 2 of germaria (C′ and D′). (E) Blocking cell death by overexpressing p35 in the germline does not rescue *pie* mutant induced GSC loss. (F) Newly emerged *w*^-^ females fed on poor-diet for 5 days (blue bars) exhibit significantly more cell death in region 2, compared to rich-dieted animals of same genotype (red bars); while overexpressing p35 in well-fed females blocked the basal frequency of cell death.

Although the function was not fully understood, *pie* was originally reported to be required for cell survival; the ectopic expression of the caspase inhibitor protein baculovirus p35 can partially rescue the rough eye phenotype (Shi *et al.* 2003). To test the possibility that *pie* mutant phenotypes in GSC maintenance are the result of cell death, we examined the cell death marker, cleaved caspase 3 in clonal germaria. On the basis of previous evidence and our observation from TUNEL assay, germline cells in region 2 serve as a checkpoint for programmed cell death, which show an increase in apoptosis under nutrient-deprived condition (Figure 4, A and B) (Drummond-Barbosa and Spradling 2001). Cleaved caspase 3 antibody specifically recognizes apoptotic cells and detects similar pattern with TUNEL in region 2 (Figure 4C) (Baum *et al.* 2007; Nezis *et al.* 2009). To our surprise, lack of *pie* function in GSCs did not result in apoptosis: we never observed activated caspase3 in either mutant or wild-type GSCs (Figure 4, C and D; Table 3). To further test whether p35 overexpression could rescue *pie* GSC loss, we expressed UASp-p35 in the germline under nos-Gal4 driver, in the *pie* clonal background and examined the *pie* GSC maintenance at days 5, 8, and 11 after clonal induction. Expression of p35 in germline was able to inhibit caspase-dependent cell death in region 2, and the whole germarium, indicated by reduced occurrence of caspase 3+ cysts (Figure 4F). However, the occurrence and loss rates of *pie^f05500^* ;nosGal4/UASp-p35 GSCs are respectively comparable with those without p35 expression (Figure 4F; Table 4), demonstrating that p35 expression does not rescue *pie* phenotype in this cell type. These data indicate that *Pie* loss-of-function induces GSC maintenance failure through other mechanisms than GSC cell death.

**Table 3:** The requirement for pie in GSC maintenance is not attributable to cell death

%Caspase3+	Clonal GSCs	Nonclonal GSCs	Anterior Clonal Cysts	Anterior Nonclonal Cysts	Posterior Clonal Cysts	Posterior Nonclonal Cysts
hsFLP; Ubi-GFP FRT40A/pie^f05500^ FRT40A	0% (n = 34)	0% (n = 109)	17% (n = 64)	1.4% (n = 218)	0% (n = 50)	0% (n = 156)
hsFLP; Ubi-GFP FRT40A/pie ^E1-16^ FRT40A	0% (n = 12)	0% (n = 68)	18% (n = 33)	8.1% (n = 111)	3.8% (n = 26)	1.2% (n = 83)
hsFLP; Ubi-GFP FRT40A/FRT40A	0% (n = 78)	0% (n = 94)	3.0% (n = 168)	8.3% (n = 218)	1.3% (n = 75)	0% (n = 157)

GFP, green fluorescent protein; GSC, germline stem cell.

**Table 4:** p35 overexpression cannot rescue pie-induced GSC loss

Genotype	Days after Induction	% of Germaria with Clonal GSC		
		Exp. I	Exp. II	Exp. III
hsFLP; pie^f05500^ FRT40A/Ubi-GFP FRT40A	5 days	27% (n = 85)	34% (n = 100)	27% (n = 62)
	8 days	7% (n = 90)	8% (n = 91)	5% (n = 80)
	11 days	3.6% (n = 55)	6% (n = 47)	1% (n = 100)
hsFLP; pie^f05500^ FRT40A/Ubi-GFP FRT40A; nosGal4/UASp-P35	5 days	25.9% (n = 116)	21% (n = 85)	/
	8 days	6.6% (n = 121)	6.3% (n = 111)	/
	11 days	3.9% (n = 76)	1.7% (n = 59)	/

GSC, germline stem cell.

### *Pie* is required for GSC maintenance through regulating BMP signaling

**Figure 5:**
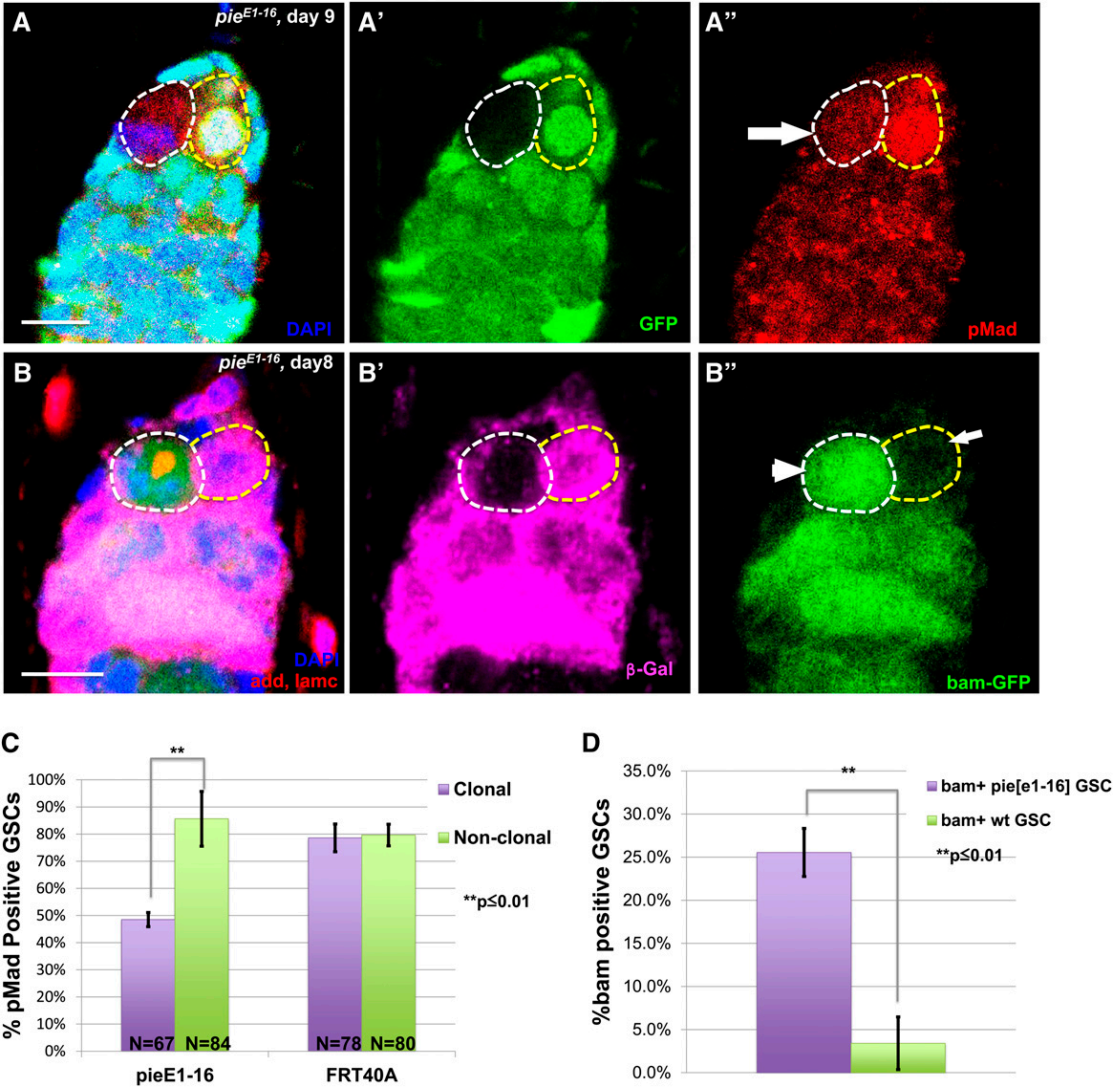
*pie* is required for GSC self-renewal through regulating BMP signaling. (A) 9 days after heat shock induction, *pie^E1-16^* mutant GSC (GFP negative, white dash circle) displays reduced nucleus pMad level compared to a wild type GSC (GFP positive, yellow dash circle). Scale bar = 10μm. (B) 8 days after heat shock induction, bam-GFP is expressed in *pie^E1-16^* mutant GSC (white dash circle, arrow head), but repressed in wild-type GSC neighbor (yellow dash circle, arrow). Scale bar = 10μm. (C) *pie^E1-16^* clonal GSCs at day 8-9 after heat shock induction have significantly smaller frequency of pMad expression compared to non-clonal neighbors or *FRT40A* control clonal GSCs. (D) *pie^E1-16^* clonal GSCs at day 8 after heat shock induction have significantly higher bam expression compared to wild-type non-clonal GSCs.

Self-renewal of female GSCs is primarily regulated by BMP signaling from the niche. Somatic niche cells express the ligands *dpp* and *gbb*, activating cytoplasmic *Mad* and *Medea*, the *Drosophila* Smads, in GSCs, which form complex and silence the transcription of the *bam* gene, the key differentiation factor that is normally turned off (Chen and McKearin 2003a; Song *et al.* 2004). Therefore, activation of this short-range BMP signal can be indicated by accumulation of BMP response gene products, phosphorylated *Mad* (*pMad*) *and Daughters against dpp* (*Dad*) primarily in the GSCs, and repressed expression of differentiation of the *bam* gene in GSCs (Song *et al.* 2004). To determine whether *pie* mutant induced GSC loss is attributable to altered BMP signaling in GSCs, we analyzed the pMad level in clonal germaria 8 to 9 days after the induction, when most of the *pie* mutant GSCs are leaving the niche. We observed a significantly lower frequency of pMad expression in *pie^E1-16^* GSCs compared with the neighboring wild-type GSCs or FRT40A control ones (Figure 5A”, arrow; C). Because of the lack of reliable antibodies against *bam*, we used a *bam-GFP* enhancer-trap allele (Chen and McKearin 2003b) to examine *bam* expression in clonal germaria. Compared with the wild-type GSCs, in which *bam* expression is mostly turned off (Figure 5B”, arrow; D), 25.9% *pie* mutant GSCs express *bam* 8 days after induction (Figure 5B”, arrow head; D), matching with the decline of pMad expression. This evidence conclusively suggests that *pie* regulates GSC maintenance through mediating with BMP signaling.

## Discussion

*Drosophila* GSCs and their niche provide a perfect model system to study adult stem cell behaviors, including essential factors and signaling pathways involved in stem cell maintenance, division, and differentiation. With this system and the powerful *Drosophila* genetics, we performed a loss-of-function screen and discovered a group of genes encoding various ubiquitin ligases in flies that are essential for GSC self-renewal. We followed up with *pie* and found that *pie* is cell autonomously required for GSC maintenance. Despite the previous finding in imaginal disc and eye tissue that *pie* mutant leads to apoptosis-related cell death (Shi *et al.* 2003), we provided evidence that *pie* loss-of-function induced GSC loss is not related to programmed cell death.

Ubiquitin-mediated protein degradation plays a variety of roles in the regulation of many developmental processes, including mediating stem cell proliferation and stem cell division through degradation of cell cycle regulators (Rathinam 2011). An E2 ubiquitin-conjugating enzyme in *Drosophila*, Effete (Eff), has been reported to regulate GSC maintenance through targeting cyclin A for degradation (Chen *et al.* 2009). Evidence from mammalian system has demonstrated that Fbxw7, an F-box protein subunit of an SCF-type ubiquitin ligase complex, targets positive regulators of the cell cycle—including Cyclin E, c-Myc, Notch, and c-Jun, and maintains mouse hematopoietic stem cell quiescent to preserve their capacity for self-renewal (Onoyama and Nakayama 2008). *pie* shares a highly similar sequence with the PHD/RING domains of human G2E3, an E3 ligase essential for mammalian early embryo development. Biochemical evidence has demonstrated that these PHD/RING domains are responsible for the catalytic function of G2E3 (Brooks *et al.* 2008). Therefore, we speculate a novel role of *pie* as an ubiquitin ligase other than a cell survival factor in *Drosophila* GSC maintenance, in a tissue-specific manner. It is possible that, as a ubiquitin ligase, *pie* might regulate targets that in one cell type involve in self-renewing division, whereas in another cell type result in survival against apoptotic cell death.

The BMP pathway is the major signaling pathway regulating *Drosophila* GSC self-renewal as well as proliferation (Song *et al.* 2004; Li and Xie 2005). Two BMP ligands, *dpp* and *gbb*, expressed in TF/cap cells, directly act on GSCs to control their self-renewal and division (Song *et al.* 2004). Activation of this short-range BMP signal is proved by the accumulation of BMP response gene products, *pMad* and *Dad* primarily in the GSC, and repressed expression of differentiation gene *bam* in GSCs (Song *et al.* 2004). We observed reduced pMad level in accord with premature expression of *bam* in *pie* mutant GSCs, suggesting a role of *pie* in the regulation of BMP signaling to maintain the stem cell identity. In contrast to *Mad*, *Dad* functions as an antagonist of *Dpp*, thus forming a negative-feedback loop of BMP signaling (Tsuneizumi *et al.* 1997). A possible mechanism would be that *pie* serves as an E3 ubiquitin ligase in GSCs, which targets Dad protein for poly-ubiquitination and degradation by proteasome. Further evidence and exploration are needed to test the hypothesis.

## Supplementary Material

Supporting Information
