# Supplementary material for: Loss-of-Function Screen Reveals Novel Regulators Required for Drosophila Germline Stem Cell Self-Renewal
Source: G3 (Bethesda). 2012 Mar 1;2(3):343–51. doi: 10.1534/g3.111.001651 (PMC3291504; doi:10.1534/g3.111.001651)
Supplement: Supporting Information [file supp_2_3_343__index.html]

Supporting Information 

# Loss-of-Function Screen Reveals Novel Regulators Required for *Drosophila* Germline Stem Cell Self-Renewal

## Supporting Information for Xing *et al*, 2012

**Files in this Data Supplement:**

- Table S1 - Alleles utilized in the loss-of-function screen (.xlsx, 63 KB)
